# Correction: Efficient *in situ* N-heterocyclic carbene palladium(ii) generated from Pd(OAc)_2_ catalysts for carbonylative Suzuki coupling reactions of arylboronic acids with 2-bromopyridine under inert conditions leading to unsymmetrical arylpyridine ketones: synthesis, characterization and cytotoxic activities

**DOI:** 10.1039/c8ra90103a

**Published:** 2018-12-21

**Authors:** Nedra Touj, Abdullah S. Al-Ayed, Mathieu Sauthier, Lamjed Mansour, Abdel Halim Harrath, Jameel Al-Tamimi, Ismail Özdemir, Sedat Yaşar, Naceur Hamdi

**Affiliations:** Research Laboratory of Environmental Sciences and Technologies (LR16ES09), Higher Institute of Environmental Sciences and Technology, University of Carthage Hammam-Lif Tunisia naceur.hamdi@isste.rnu.tn +966-6333-9351 +966-556-394-839; Chemistry Department, College of Science and Arts, Qassim University Al-Rass Kingdom of Saudi Arabia; Ecole Nationale Supérieure de Chimie de Lille, Unité de Catalyse et Chimie du Solide, UMR CNRS 8181, USTL BP 90108 59652 Villeneuve d’Ascq France; Zoology Department, College of Science, King Saud University P.O. Box 2455 Riyadh 11451 Saudi Arabia; İnönü University, Faculty of Science and Art, Department of Chemistry Malatya Turkey

## Abstract

Correction for ‘Efficient *in situ* N-heterocyclic carbene palladium(ii) generated from Pd(OAc)_2_ catalysts for carbonylative Suzuki coupling reactions of arylboronic acids with 2-bromopyridine under inert conditions leading to unsymmetrical arylpyridine ketones: synthesis, characterization and cytotoxic activities’ by Nedra Touj *et al.*, *RSC Adv.*, 2018, **8**, 40000–40015.

Errors were present in the published article in terms of the co-author name spelling for J. Al-Tamimi and the author affiliations. The corrected list of author and affiliation details in this paper is as shown here.

The Royal Society of Chemistry apologises for these errors and any consequent inconvenience to authors and readers.

## Supplementary Material

